# Fabrication of Polypill Pharmaceutical Dosage Forms Using Fused Deposition Modeling 3D Printing: A Systematic Review

**DOI:** 10.3390/pharmaceutics16101285

**Published:** 2024-09-30

**Authors:** Haya Yasin, Moawia M. A. Al-Tabakha, Siok Yee Chan

**Affiliations:** 1 School of Pharmaceutical Sciences, Universiti Sains Malaysia, Gelugor 11800, Pulau Pinang, Malaysia; haya.yasin@gmail.com; 2 Department of Pharmaceutical Sciences, College of Pharmacy and Health Sciences, Ajman University, Ajman P.O. Box 346, United Arab Emirates

**Keywords:** 3D drug printing, fused deposition modeling, drug manufacturing, dosage form, drug delivery, personalized medicine

## Abstract

Background/Objectives: The pharmacy profession has undergone significant changes driven by advancements in patient care and healthcare systems. The FDA approval of Spritam^®^ (levetiracetam), the first 3D-printed drug, has sparked increased interest in the use of Fused Deposition Modeling (FDM) 3D printing for pharmaceutical applications, particularly in the production of polypills. Methods: This review provides an overview of FDM 3D printing in the development of pharmaceutical dosage forms, focusing on its operation, printing parameters, materials, additives, advantages, and limitations. Key aspects, such as the ability to personalize medication and the challenges associated with the technique, including drug stability at high temperatures, are discussed. Results: Fourteen studies relevant to FDM 3D-printed polypills were analyzed from an initial pool of 60. The increasing number of publications highlights the growing global interest in this technology, with the UK contributing the highest number of studies. Conclusions: FDM 3D printing offers significant potential for personalized medicine by enabling precise control over dosage forms and tailoring treatments to individual patient needs. However, limitations such as high printing temperatures and the lack of standardized GMP guidelines for large-scale production must be addressed to fully realize its potential in pharmaceutical manufacturing.

## 1. Introduction

**Figure 1:**
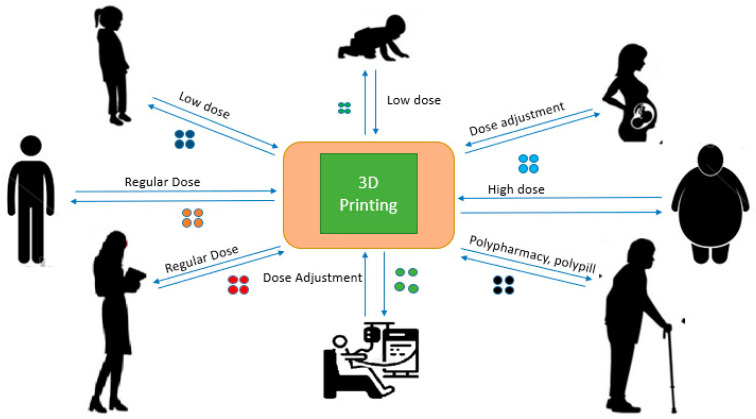
Personalized medication to fit different patient needs.

Over the last few decades, there have been abrupt changes in the medical field; it constantly evolves based on the modern needs of patients and the healthcare system [1,2], as there has been a growing emphasis on patient-centered care and evidence-based medicine. The dynamic nature of the medical field is driven by a combination of scientific progress, societal needs, patient expectations, and policy changes, all of which contribute to its constant evolution to meet the modern needs of patients and the healthcare system. As patients and healthcare systems become more intricate, the demand for tailored medication is increasingly vital to enhance patient care and achieve better health outcomes [3]. In this regard, personalized medicine overcomes the “one-size fits all” concept for the treatment and care of particular conditions in patients [4]. For example, for patients with problems in metabolism (too low or too high) and patients taking various drugs that may interact, as well as in pediatrics and geriatrics, customized dosing is preferred. Extemporaneous preparation, therapeutic drug monitoring, polypills, and phenotyping are among the most recent solutions for personalized medicine Figure 1 [5].

Three-dimensional (3D) printing technology is a highly propitious technique for tailoring dosage forms based on the patient’s need. It requires a simple machine with fewer steps and a small operational space, therefore making it easier and more efficient compared to the conventional production methods [6]. This enables the control of dosage forms, the number of drugs incorporated in the dosage form, and the amount of each drug incorporated based on the patients’ needs. With the first FDA-approved drug, Spritam^®^ (levetiracetam), manufactured by Aprecia Pharma, Inc. Blue Ash, Ohio, USA. using 3D Fused Deposition Modeling (FDM) printing, the research on 3D printing has increased for the fabrication of dosage forms in general [7,8,9].

The term FDM, “Fused Deposition Modeling”, is based upon extrusion, where it relies on building multiple layers of melted material [10]. In contrast to other 3D printers, Fused Deposition Modeling (FDM) requires a reduced number of post-processing procedures, thereby improving production efficiency and resulting in time and cost savings. As a result, FDM has the potential for small-scale production and dose fabrication in 3D printing technology [11,12,13,14,15].

FDM 3D printers are found in the market at a low cost, are portable, easy to use, and have high resolution and precision, making them the most eligible 3D printers to be implemented in the new era of compounding pharmacies [16,17]. FDM 3D printers allow easy control of constituents (APIs and polymers by HME), type of dosage form, design, dosage flexibility, and variability to meet the needs of the patient based on their age, background, weight, metabolism, body surface area, height, and disease conditions. In contrast to traditional manufacturing, FDM 3D increases the opportunity for drug development, testing, and manufacturing medications with different shapes, sizes, and shell thicknesses with unique release rate behavior. The ease of control of printing parameters, such as the infill percentage of dosage, shape, density, and ability to form multi-drug systems with complex release profiles, fulfills the needed release profiles of the final printed dosage forms. The ability of on-demand production will overcome stability issues and decrease medications going to waste due to expiry dates. FDM on-demand 3D printing will allow the production of dosage forms at a lower cost.

As the nature of the FDM 3D printing method involves manufacturing and distribution at the same place, this decreases the need for a distribution and storage system and, hence, reduces the costs associated with packaging, prescribing, dispensing, and storage. Drug product shortages will also no longer be an issue, particularly for patients without an alternative treatment strategy [18].

Rapid changes in the field of healthcare, along with the increase in the complexity of patient cases, underline the need for innovative strategies, especially for the prevalence of co-existing conditions and polypharmacy [19]. In this regard, up to six different medications could be incorporated into a single tablet by 3DP, also known as a polypill [20]. Briefly, polypills are a single dosage form containing more than one active pharmaceutical ingredient. Drug dosing could be modified and adjusted by the chosen physical parameters of the designed dosage form or polymer choice by altering the drug loading percentage of the intended dosage forms or by tailoring various aspects of the formulation (e.g., compartments, flavor, shape, color) [21]. All these factors will achieve better therapeutic outcomes and increase patient compliance [22,23,24,25].

While there has been enormous progress in the field of FDM 3DP, only a handful of studies have revealed the polypill development of FDM 3D printing. Review articles in the FDM 3D field have mainly examined the operational principles of Hot Melt Extrusion and Fused Deposition Modeling, along with their potential synergies for advanced pharmaceutical applications [16]. Abaci et al., in 2021, offer an in-depth review of Additive Manufacturing (AM) methods and materials used in the production of oral tablets, addressing the challenges associated with pharmaceutical formulations and suggesting potential solutions [26]. Furthermore, Dumpa et al., in their 2021 review, explored the stages of FDM 3D printing, which encompassed feedstock filament preparation using HME, digital dosage form designs, filament characterization, various novel applications, and future prospects. Overall, there remain gaps in the overview of polypill-associated extrusion and printing models, the printing process, and the delineation of the inclusion of multiple active pharmaceutical ingredients [27].

Therefore, this review aims to examine FDM 3D printing technology, its extrusion and printing processes, and the variables affecting each stage. It provides a detailed analysis of printed dosage forms containing more than one API and their extrusion and printing parameters. Specifically, polypill dosage forms will be the focus, with a systematic discussion of challenges, advantages, limitations, and the future potential of FDM 3D printing in the pharmaceutical field. This review follows the PRISMA (Preferred Reporting Items for Systematic Reviews and Meta-Analyses) methodology to provide a structured framework for utilizing FDM 3D printing in polypill production.

## 2. Materials and Methods

### 2.1. Inclusion Criteria

Original research publications encompassing the production of polypills using FDM 3D printing are included in the current review paper. General articles on 3D printing are also included. All publications included were written in English.

### 2.2. Exclusion Criteria

Research publications on printed dosage forms with no extruded API were excluded. Research publications on 3D printing using different techniques or where the main focus theme was not FDM were also excluded. The main focus was to determine whether the FDM printer was used in the research to produce a printed dosage form with multiple active ingredients.

### 2.3. Search Strategy

**Figure 2:**
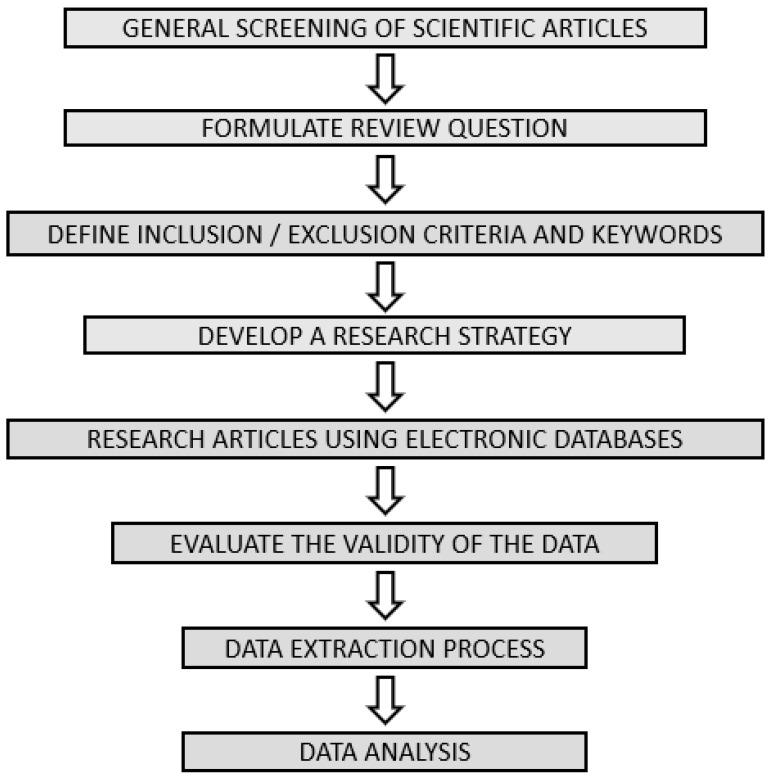
Steps to conduct a PRISMA review.

The search strategy was based on the Preferred Reporting Items for Systematic Reviews and Meta-Analyses (PRISMA) guidelines PRISMA registration number: https://doi.org/10.17605/OSF.IO/WHQST (accessed on 19 September 2024). The search phrases used as input in Pubmed and Scopus consisted of one or different combinations of the keywords. The keywords used are as follows: “three-dimensional printing”, and “3D”, and “Fused Deposition Modeling”, and “FDM”, and “multiple API”, and “polypill”, “combipill”. When available, the “Cited by” and “Similar articles” sections were also considered. The needed changes to the keywords were made, as systems do not index all terms in the same manner. No limitations in regard to the year of publication were made. In July 2024, the search was concluded, and a flowchart for the review is presented in Figure 2. Data from each report were reviewed by a minimum of two independent reviewers. This dual-review approach helped to enhance the accuracy and consistency of the collected data. The reviewers worked independently to minimize bias. To ensure the validity of the review’s conclusions, it is crucial to assess the risk of bias in each included study. To evaluate the risk of bias in the included studies, we utilized the Cochrane Risk of Bias Tool, which assesses domains such as selection bias, performance bias, detection bias, and attrition bias. Two independent reviewers applied the tool to each study. Any discrepancies in assessments were resolved through discussion or with input from a third reviewer. This review was not registered with any official register.

### 2.4. Literature Search Output

**Figure 3:**
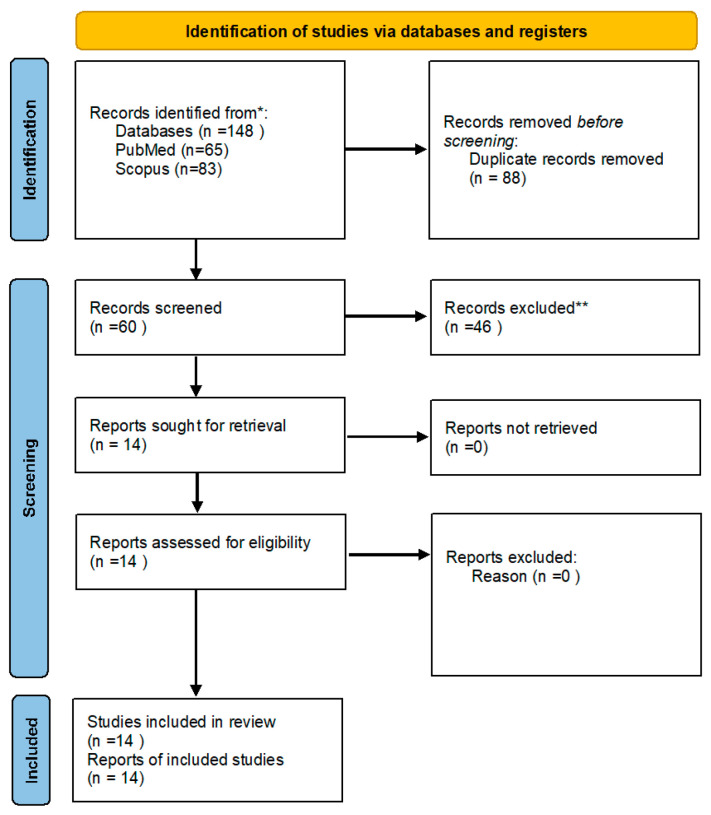
Flowchart of literature search output. * duplicate records, ** excluded for not focusing on FDM, or single API.

The total outcome of the search was 148 articles. Duplicates were removed, resulting in 60 unique articles. Records were screened, and articles were excluded for not focusing on FDM (*n* = 28) [20] or a single active ingredient was used in the dosage form (*n* = 18) [28], resulting in 14 publications included in this review. The flowchart of literature search output illustrated in Figure 3. The increased number of publications every year highlights the growing interest in FDM printing. It was noted that FDM studies are primarily carried out in certain countries, with approximately 16% in the United Kingdom (UK), which had the highest number of articles published.

## 3. Fused Deposition Modeling Printing Principle and Setup

**Figure 4:**
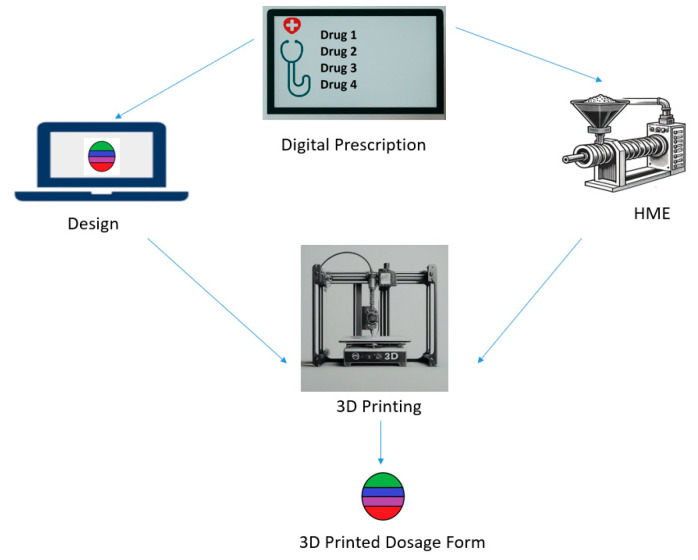
Hot Melt Extrusion (HME) coupled with FDM for polypill fabrication.

3D printing technologies operate in a cohesive manner. First, the model is created using a computerized aided design, “CAD”, magnetic resonance imaging scan, or computerized tomography. The created design is read by the 3D-printer software in (STL) file format. Then, the processing and printing criteria are set, and the 3D-printer software will slice the model into a layered structure. Finally, the product is printed layer by layer and evaluated [29,30]. In the following sections, the FDM technology technique for 3D printing of pharmaceutical dosage forms is considered. Section 7 provides a comprehensivelist of printed dosage forms with more than one API with extrusion and printing details. Figure 4 illustrates Hot Melt Extrusion (HME) coupled with FDM for polypill fabrication.

### 3.1. Creating the 3D Digital Pharmaceutical Dosage Form

The first step of the 3D printing process is creating the structure of the desired pharmaceutical dosage form according to the necessities of the medication. Shape (capsules, tablets, implants, films, lozenges), fills (hollow, porosity, density), thickness, and the number of compartments are created using “CAD” software. The choice of software depends on the complexity of the design. From our research, the most commonly used CAD software for modeling the 3D-printing of polypills are Autodesk Fusion 360^®^ Autodesk Inc. (San Rafael, CA, USA), and Autodesk AutoCAD^®^ Autodesk Inc. (San Rafael, CA, USA). Other used software are Creo^®^ PTC Inc. (Needham, MA, USA), Solidworks^®^ Dassault Systèmes, Vélizy-Villacoublay, France Rhinoceros^®^ Robert McNeel & Associates (Seattle, WA, USA) and Simplify3D ^®^ Simplify3D, LLC (Columbus, OH, USA).

### 3.2. Transforming 3D Model into a 3D Printer-Readable Version

After creating the computerized 3D model, the format file of the CAD has to be converted to a readable file for 3D printing, an STL file format [31]. In literature, the most commonly used software for the production of polypills using FDM are Simplify 3D^®^, Simplify3D, LLCand Maker ware^®^, MakerBot Industries, LLC, New York, NY, USA.

### 3.3. Fixing Process Parameters and Slicing the Model Layer by Layer

The STL file is fed into the FDM software of the 3D printer. The FDM software will slice the created CAD design into a layer-by-layer pattern and send it to the 3DP to print the dosage form. Ensuring the stability of the process parameters is equally crucial since it will regulate the three-dimensional structure of the ultimately printed pharmaceutical dosage form. Slicing software controls tell the printer how to print according to the process parameters. Depending on the final pharmaceutical dosage form product and the needed properties and application, the process parameters are set. Some of the process parameters are size and geometry (length, width, and height); infills (density, pattern, and wall thickness); printing parameters, such as speed, nozzle and bed temperature, and pressure; and number of nozzle heads [32]. All the mentioned parameters can be modified and enhanced to reach the desired dosage form product [33,34]. Further discussion of factors affecting printing is presented in Section 5.

### 3.4. Fabrication and Formulation of Filaments

Finding the right filament and the needed properties is highly important in the 3D printing process. The filament should have the right consistency and thermal properties to be fed into the 3D printer. Prior to feeding the filament to the 3D printer, the API, additives, and carriers are added to the filament. The needed ingredients are added to the filament using the solvent immersion method or the Hot Melt Extrusion method [35]. API incorporation into the pharmaceutical-grade polymer filaments for 3DP by several techniques is further discussed in Section 6.2.

### 3.5. Printing and Evaluation of the Final Printed Dosage Form

The FDM 3D printer uses heat to melt the filament. The melted polymer is set by the nozzle head layer by layer in three directions (*x*, *y*, and *z* axes), solidifying and binding to the layer beneath and creating a final 3D pharmaceutical dosage form [36]. The FDM can contain one or more print heads, which are controlled independently, allowing the extrusion of several materials, which is controlled by the CAD model and printer parameters.

## 4. Incorporating Active Pharmaceutical Ingredients

Incorporating API into filaments presents a significant challenge. This particular step poses a significant challenge due to one of the constraints of FDM, which is the restricted drug capacity [11,37]. Since ready-made filaments with incorporated drugs are not commercially available [38], a step of incorporating the drug into a pharmaceutical-grade polymer prior to printing is mandatory. The primary method for loading drugs into filaments is “Hot Melt Extrusion coupled with FDM”.

### 4.1. Solvent Immersion

In this process, the filament is usually soaked in a prepared solution or suspension containing an active pharmaceutical ingredient, where the API is incorporated into the polymer by a diffusion process. The passive diffusion process of the API into the polymer in the filament or tablet is time-consuming and results in a low drug loading percentage [37]. The loading percentages depend on the solubility of the drug in the immersed solution or suspension [11,39]. This process was used to produce the first filament for FDM. Whilst this process is easy and simple, due to the major limitation of drug loading, it is less favorable. This process was the least favorable amongst scientists; approximately 10% of articles used this method for FDM-printed dosage forms, whilst in polypill production, no literature was found using the solvent immersion method [11].

### 4.2. Hot Melt Extrusion (HME) Coupled with FDM

The HME process is the more favorable method for filament fabrication. It produces a more homogenous drug-loaded filament [40] with higher drug amounts and dosing flexibility [41]. HME allows for the formation of filaments with good mechanical and rheological properties [42,43,44]. API, plasticizer, lubricants, excipients, and the pharmaceutical-grade polymer are ground and mixed thoroughly to allow the constituents to have similar particle sizes, which will ensure a homogeneous filament. 

The properties of the API, such as its solubility and melting point, will control whether the drug will disperse, dissolve, or melt in the pharmaceutical-grade polymer [45]. The homogenous mixture will then be fed to the feeder in the extruder. The extruder, which is the main component, usually consists of a feeder, a motor, rotating screws (single or double), an extrusion barrel, a heater, and a die [46]. The heaters convey heat to melt the ingredients, and the screws produce stress for strong mixing of the ingredients. Both the heat and the stress allow materials to melt. The screw then forces the melted material down to the barrel and the die. The die will form the shape of the final filament. 

Some of the parameters that can be controlled in the extrusion process are the feed rate, heating temperature, screw speed in revolutions per minute (RPM), and the vacuum level [46]. Compared to the solvent immersion method, HME has several advantages over solvent immersion. HME can be considered a solvent-free process; therefore, no drying step is needed. Another advantage is the higher amount of drug in the drug-loading filament produced, usually in the range of 5–40% [47,48]. The continuous production with fewer processing steps and better rheological properties of the final filament are also major advantages [49,50]. HME requires pressure and heat; therefore, the thermal degradation of API during the process is a major challenge. Plasticizers, lubricants, and excipients should be considered in the extrusion process. Gioumoxouzis et al., studied the difference in filament production and the effect on the polypill containing metformin and glimepiride between using a twin-screw extruder, HAAKE MiniLab^®^, (Thermo Fisher Scientific, Waltham, MA, USA) and a single-screw extruder, Filabot^®^ (Barre, VT, USA). The twin-screw extruder required higher temperatures, around 20 degrees Celsius, to extrude the same mixtures in comparison to the single-screw extruder [51].

Polypills produced from the twin-screw extruder showed slightly slower metformin release. This can be explained by the slightly bigger filament produced by the twin-screw compared to the single-screw extruder [52]. Rycerz et al. studied the effect of API concentration on the extrudability of the filament. The increase in the concentration of API in the suspension, leading to excessive viscosity, resulted in it being non-extrudable [53]. Sadia et al. studied the concentration of HCT by varying the drug concentration from 2.5% to 50% *w*/*w*, as follows: HCT: EM—25:5, 25:10, 25:20, 12.5:5, 12.5:10, or 12.5:20 mg:mg. A decreasing HCT ratio in the filament led to a lower degradation percentage at elevated temperatures (>400 °C), t. This was attributed to filaments of low HCT concentrations containing a significant amount of the thermally stable filler (TCP) [54]. Around 70% of research articles used HME coupling with FDM to produce dosage forms, and 83.3% of research articles used HME coupling with FDM to produce polypills.

### 4.3. Print and Fill

This technique involves printing a vacant outer shell and filling it manually with API. API can be in liquid, semi-solid, or powder form. The outer shell is made of fabricated filaments from pharmaceutical-grade polymers that control the dissolution release profiles and kinetics of the final 3D-printed dosage form [43]. Printing and filling can be either concurrent or subsequent. In the first scenario, printing and filling are conducted simultaneously, whilst in the second, the printing is paused, API is filled, then printing resumes, and the shell is closed [44]. The printing can also be done in separate parts, which will be joined after filling [45]. Around 19.9% of research articles used the print-and-fill process to produce FDM-printed dosage forms, and 16.7% of research articles used print-and-fill to produce polypills.

## 5. Printing and Extrusion Parameters

Processing parameters of FDM can influence the quality, effectiveness, and performance of the final printed dosage form. Studying each processing parameter and its effect on product quality is of great importance for manufacturing a final printed dosage form with the required characteristics [54]. Processing parameters such as printing temperature, filaments, build platform temperature, printing speed, printing temperature, nozzle size, printing dimensions, infill density, infill pattern, shell thickness, top and bottom thickness, and layer height are discussed in this section.

### 5.1. Printing and Extrusion Temperature

One of the main parameters with FDM 3DP is the printing temperature. The temperature should suitably melt the constituents and achieve the desired viscosity to flow through the printer and deposit layer by layer in a timely manner. Setting the right temperature depends greatly on the melting temperature of the API, polymer, and excipients, ensuring those mentioned are not affected by degradation. Using printing temperatures above the melting or glass transition temperature of the used polymer during printing is important to ensure adequate flow and avoid blockage of the nozzle [55]. Keikhosravi et al., 2020 created a polypill with two compartments using FDM. Eudragit^®^ L100-55, Rahavard Tamin Pharmaceutical Company (Tehran, Iran) was utilized in the preparation of the polypill, which contained aspirin and simvastatin. The researchers investigated the impact of different extrusion temperatures on the pill. They found that a lower temperature of 135 °C reduced the risk of polymer degradation during the HME process [56]. Kulkarni et al., 2022 studied the effect of different extrusion and printing temperatures of 150 °C and 210 °C on the final polypill produced by FDM 3DP; discoloration of the filament was observed on the higher temperature, while no difference was observed in the printing quality. Hence, the printing was conducted at the lowest temperature possible [57]. Three-dimensional printing occurs in a layer-by-layer manner. The printed product has to stay in contact with the building platform at a specific temperature to adhere to printed layers and achieve the final dosage form. Insufficient build plate temperature will cause the dosage form to lose contact with the platform, interrupt printing, and result in an incomplete dosage form [58]. 

### 5.2. Printing Speed

Printing speed is fundamental to print accuracy and precision. The speed controls the accurate feeding of the molten material and precise layer-by-layer deposition, avoiding detachment or jamming. A lower printing speed may be preferred to overcome these issues, taking into consideration the time needed to print a single dosage form [38]. In a study conducted by Krishna Mohan et al., an increase in printing speed from 30 mm/s to 65 mm/s decreased the deviation from 8.57% to 4.5%, which suggested an increase in print speed increases dimensional accuracy [52,59], and studied the effect of printing speed on pediatric soft dosage forms containing paracetamol and ibuprofen. The printing patterns were assessed at the following needle speeds: 50, 55, 60, or 65 mm/min. Reducing the speed of printing generally resulted in an increase in the amount of dispensed paracetamol using the same printing patterns. This was attributed to extruding larger amounts of material with a longer time available to complete the shape [52].

### 5.3. Nozzle Size

Nozzle size controls various aspects of printing. Larger nozzles print faster with fewer details, whilst smaller nozzles print slower with greater details and resolution. Larger nozzles result in higher mechanical properties than smaller nozzles due to the formation of macrostructures with higher degrees of interconnection layers and paths [60]. Nozzles with small diameters are to be handled carefully, as they may become clogged with the melted constituents [61]. A smaller nozzle diameter achieves a more favorable and better dimensional accuracy and surface roughness, resulting in an increase in the printing time. Blocked nozzles are a major concern for the printing of dosage forms, as they affect the quality and accurate dosing of pharmaceutical dosage forms [62,63]. Rycerz et al., 2019 also studied the effect of nozzle size on the final printed dosage form containing paracetamol and ibuprofen. Nozzle sizes G15 (1.52 mm), G16 (1.34 mm), and G17 (1 mm) were used. The larger the nozzle size used, the higher the dose of paracetamol extruded. The higher doses of paracetamol were extruded with reduced resistance. Therefore, G15 (1.52 mm) was chosen as the default to print the final dosage form [52]. 

### 5.4. Printing Dimensions and Design

The printing dimensions control the shape and size of the final printed dosage form. The size and shape can influence the disintegration and release rate of the API. Several researchers studied the effect of the size and shape of the printed dosage form. Goyanes et al. printed several shapes (sphere, ring, cylinder, or caplets) [64,65] to investigate the kinetics of each printed shape. The dissolution rate increased as the surface area to volume ratio increased [66]. Windolf et al. studied the effect of the polypill design on the dissolution of the final printed polypill tablet. A simple polypill design was chosen (PP1); a cylinder with a diameter of 10 mm was selected as the geometry, which should, therefore, be easy to swallow. To increase the dose, a hollow cylinder geometry (PP2) was designed and built up in three layers. Compared to PP1, PDM is released more slowly here. This is due to the enclosed SA of the two LD/BZ hollow cylinders. Due to the small outer SA of the PVA formulation in contact with the medium (24% of the SA), the separation of the layers could not proceed as quickly as desired, so the increase in SA due to the separation of the hollow cylinders occurred late and, thus, did not lead to a faster API dissolution. The PP3 design was a hollow cylinder, this time with a small cylinder inlay printed; API is quickly released from the matrix. Due to tablet sizes not being feasible for patients with swallowing difficulties, mini tablets were investigated. Minitab and mini hollow cylinder (minihc) were investigated; the mini hollow cylinder (minihc) had a higher release rate [67].

### 5.5. Infill Density

Infill density is how full the inside part of the printed dosage form is. It is usually defined in percentages from 0 to 100%; at 0% percent, the printed dosage form will be hollow, whilst at 100%, completely solid. The infill density is an important factor that has a high impact on the drug release kinetics and the mechanical properties of the final printed dosage form. The higher the infill density, the harder the final dosage form, resulting in lower disintegration and a lower drug release rate [68]. Wang et al. studied the effect of different infill densities on the drug release rates [69]. The polypills containing caffeine and vitamin B were fabricated with various infill densities, including 50%, 75%, and 100%. An increase in release rate was observed as the infill density decreased. Sustained release is realized by adjusting the release profiles and regulating the infill density of the different printed layers.

The investigation conducted by Zhang et al. focused on examining the influence of the geometrical design and material compositions of combination pills of tranexamic acid and indomethacin on the rate of drug release; 30% infill and 10% infill FDM polypills were investigated [70]. With 10% infill, more than 12% of the drug was released within the first 120 min. In contrast, only about 3.5% of the drug was released from the samples with 30% infill. The drug release rate increased with increasing pore width (lower infill density) [70].

### 5.6. Infill Pattern

The infill pattern regulates the amount of material deposited on the surface of the printed dosage, the build time, and the strength of the FDM-printed dosage form. Infill patterns can influence the porosity, rigidity, disintegration time, and in vitro release kinetics of the final printed dosage forms. The infill pattern is proportional to the strength of the dosage form; the denser the pattern, the stronger the dosage form. The infill pattern of the printed dosage form can control the dose and release kinetics of the APIs without altering the composition [37,47,71]. Pereira et al., 2020 configured the compartments of a polypill containing lisinopril, indapamide, rosuvastatin, and amlodipine in two designs, parallel or concentric, to achieve different release patterns. For extended and delayed release profiles, the concentric capsule was designed. The internal and external compartments were also studied; internal compartments of 2 mm size were used for the immediate release profile, whilst external compartments were designed to extend the drug release [72]. Another study by Periera et al. on the same drugs studied variations in the drug contents in the multilayer structure. Polypill I contained lisinopril, indapamide, rosuvastatin, and amlodipine, top to bottom. Polypill II contained rosuvastatin, amlodipine, lisinopril, and indapamide, top to bottom. A unimatrix polypill tablet with the same dimensions and drug doses was also fabricated from a single filament. Multilayer I was 3D-printed with the external layers of the most soluble drugs in an aqueous medium containing amlodipine besylate and lisinopril dihydrate, while the internal layers consisted of the two least soluble drugs, indapamide and rosuvastatin calcium. The Multilayer II structure contained the two least soluble drugs as external layers and the most soluble as internal layers.

The results of the study concluded that the unimatrix polypill drug release was slower in comparison to the individual tablets; this was attributed to the smaller surface area-to-mass ratio of the unimatrix tablet in comparison to individual drug tablets. In Multilayer polypills I and II, the release profile of each drug was dependent on the position of the layer. The results showed a biphasic drug release, as the water penetration is different for the outer drug layers compared to the inner ones, with an initial slow dissolution of some drugs that became faster after a lag time of 15 min.

In the Multilayer II format, rosuvastatin and indapamide dissolved faster than in Multilayer I; this finding highlights the significance of optimizing the order of the drug layers to achieve a desirable drug release from a multilayer tablet design. This multilayer approach offers high dose titration flexibility and allows for controlling the release of drugs of different physicochemical characteristics [73]. The unimatrix printed continuously and had more uniform layers on its surface. The drug content was expected to be similar, but that was not the case. The variations in drug content for the multilayer were explained by the deviations in the weight of each separate layer produced during the printing process. Drug release was slower in the unimatrix compared to the other tablets due to the smaller surface area-to-mass ratio of the unimatrix tablet (polymer-rich matrix). In multilayer tablets, the release profile of each drug was dependent on the position of the layer and distinctively different from the release obtained from the unimatrix tablet due to the access to water being different for the outer drug layers compared to the inner ones [73,74]. Rycerz et al., 2019 also studied the printing pattern of 25%, 50%, and 75% in pediatric soft dosage forms containing ibuprofen and paracetamol. The study concluded that the extrusion of higher doses with a larger amount of material at the same flow rate and with a longer time available to complete the shape results in good linearity for both paracetamol and ibuprofen being achieved between the percentage of printed patterns and the achieved doses [54]. Goyanes et al. produced two different capsule-shaped tablets, or “Caplets”, one being multilayered and the other being a two-compartment caplet. The multilayered caplet is composed of two back-to-back layers, each layer composed of a different drug (paracetamol or caffeine), whilst the two-compartment caplet is a small caplet inside a larger caplet—a “Duocaplet”. Two Duocaplets were produced: a Duocaplet of 9.5% caffeine-PVA (outer) and 8.2% paracetamol-PVA (core); and a Duocaplet of 8.2% paracetamol-PVA (outer) and 9.5% caffeine–PVA (core). The drug release study shows that for the multilayer device, drug release rates were similar for both drugs. For the Duocaplet, the drug on the external layer is released first. The time lag of the release of the drug in the inner caplet depends on the characteristics of the external layer. The study has proven that drug dissolution can be modulated by printing dosage forms with different shapes by FDM 3D printing, which is challenging to manufacture by the traditional production method [68]. Yufei Chen et al. studied the effect of printing patterns on the final printed dosage form; a triangular pattern showed the largest pore sizes formed (>500 μm), followed by a grid pattern (500 μm) and a rectilinear pattern (250 μm). The wiggle pattern formed triangular porous structures, while fast honeycomb and full honeycomb patterns formed irregular pores on the surface, and the grid pattern demonstrated the most uniform porosity and most consistent daily release [75].

### 5.7. Shell Thickness

With 3D printing technology, the shell or outer wall thickness can be controlled. Shell or outer wall thickness has a different infill pattern than the core of the dosage form. Final printed dosage forms without an outer shell have a rougher surface compared to dosage forms with shells [44,76]. The higher the shell thickness, the denser and thicker the final printed dosage form structure compared to without a shell. The increase in density and thickness of the shell will cause a delay in water permeation and, therefore, drug diffusion, resulting in extended drug-release properties [77,78].

Alayoubi et al. produced a polypill containing Atorvastatin, Carbopol, and Metoprolol and consisting of mesh-shaped layers. The results indicated the number of shell layers in the polypill compartment was critical for achieving the desired dissolution and drug release of the polypill. In general, the number of shell layers controlled diffusional transport by affecting shell thickness, porosity, pore distribution, diffusional path length, and tortuosity. This study demonstrates the foundational concepts for understanding the effects of critical formulation (drug and polymer loadings) and geometrical parameters (number of shell and septum layers and porosity) on the performance characteristics (drug strength, dosage uniformity, drug release, and breaking strength) of polypills to meet the needs of each individual patient [79].

## 6. Materials for 3D Printing

The added constituents and structural design greatly affect the properties of the final dosage form. Therefore, the choice of API, polymer, and excipients should be studied thoroughly. There is a need to emphasize that excipient functionality may be different in FDM than in conventional methods. For example, stearic acid in direct compression acts as a lubricant, whilst in FDM 3D printing, it works as a plasticizer in the production of theophylline tablets with Eudragit [47]. Additives should be compatible with the API and polymer, inert, inactive, and physically and chemically stable. Although not necessary, additives may be added to improve the printing process (plasticizers, binders, fillers, and lubricants), while others, e.g., release modifiers, are added to optimize the API dissolution in the final printed dosage form [80]. In this section, the significance of each constituent—API, polymers, plasticizers, binders, fillers, lubricants, release modifiers, disintegrants, and stabilizers—is discussed.

### 6.1. API

The incorporation of active pharmaceutical ingredients (APIs) in FDM 3D printing plays a vital role in the development of personalized medicine and innovative dosage forms. FDM 3D printing allows for precise control over drug loading, release profiles, and the spatial distribution of APIs within a formulation, enabling customized therapies tailored to individual patient needs. In this context, the selection and behavior of the API, particularly its physical state (amorphous or crystalline) and solubility, significantly impact the final product’s performance [81].

Hydrophilic APIs are compatible with water-soluble polymers like hydroxypropyl methylcellulose (HPMC) or polyvinyl alcohol (PVA). Hydrophilic APIs tend to have good miscibility with these types of polymers, which can help in achieving uniform drug distribution during the extrusion and printing processes [82,83,84].

Hydrophobic APIs can be incorporated using more hydrophobic polymers like polyethylene glycol (PEG), ethyl cellulose (EC), or polyvinylpyrrolidone-vinyl acetate (PVP-VA). Such APIs may require higher extrusion temperatures, but adjustments to the polymer’s melting point or incorporating plasticizers can help make them compatible with FDM [83,84,85].

In FDM 3D printing, APIs often transition to an amorphous state due to the high temperatures involved during the extrusion process. This transformation is critical, especially for poorly water-soluble drugs, as the amorphous form typically exhibits improved solubility and dissolution rates compared to the crystalline form. The thermal processing involved in FDM disrupts the crystalline lattice of the drug, resulting in a less ordered amorphous structure that enhances its dissolution and bioavailability [40,46,86].

Amorphous solid dispersions are often formed when APIs are combined with polymeric carriers, such as hydroxypropyl methylcellulose (HPMC), polyvinyl alcohol (PVA), or Kollidon VA64, BASF (Ludwigshafen, Germany). These carriers not only aid in the formation of amorphous APIs but also prevent recrystallization during storage by stabilizing the amorphous structure. The transformation to an amorphous state can be confirmed through various analytical techniques, with X-ray diffraction (XRD) being a primary method. In post-printing analysis, XRD patterns often show broad halos instead of sharp peaks, indicating the absence of crystalline structures [87].

The improved solubility and dissolution rate of amorphous APIs directly contribute to enhanced bioavailability, a significant benefit in drug formulations produced through FDM 3D printing. Many poorly soluble drugs struggle to achieve therapeutic plasma concentrations when administered in their crystalline form. By converting the drug to its amorphous state, the solubility-limited absorption can be overcome, which is crucial for achieving the desired therapeutic effect [32,81].

The process of amorphization during FDM 3D printing typically occurs during the heating and extrusion of the filament, where temperatures surpass the drug’s melting point. This rapid heating and subsequent cooling results in the API adopting an amorphous structure. However, to ensure the amorphous API remains stable and does not recrystallize, the selection of excipients, such as plasticizers and stabilizers, is essential. Excipients like polyvinylpyrrolidone (PVP) and Eudragit polymers are often added to prevent recrystallization and enhance the stability of the amorphous form [88].

The conversion of crystalline APIs to their amorphous counterparts in FDM formulations not only improves solubility but also allows for tailored drug release profiles. The amorphous state of the API facilitates rapid dissolution, which is often desirable in immediate-release formulations. However, through the strategic use of polymers and excipients, controlled-release profiles can also be achieved, allowing for a customizable approach to drug delivery [85,89].

Several studies have demonstrated the successful use of amorphous APIs in FDM 3D-printed formulations. For example, Jamróz et al. (2018) reported the use of FDM to prepare polypills with multiple APIs in an amorphous form, resulting in improved dissolution rates and therapeutic efficacy [90]. Similarly, Repka et al. (2018) highlighted the use of FDM technology to convert poorly soluble drugs into amorphous dispersions, significantly enhancing their bioavailability [84].

### 6.2. Polymers

Polymers are important excipients in 3D printing; they improve the function and delivery of the APIs. The selection of the polymer depends on the API, method of printing, and intended dissolution profile. Polymers should be compatible, inert, stable, and printable. The most commonly used polymers are polyvinyl alcohol, cellulose-based polymers, ethyl cellulose, polyvinylpyrrolidone, polycaprolactone, Carbopol, and polyethylene glycol. Polymers can be classified based on printing process temperature and release profiles. The polymer grade has an effect on the release rate, as well. In literature, the highest number of publications in FDM 3DP used a printing temperature between 100 °C and 150 °C, whilst printing temperatures < 100 °C to formulate final dosage forms were the least found in the literature; this may be due to printing at low temperatures resulting in poor adhesion and lower mechanical properties of the printed dosage forms of caffeine [91], paracetamol, metformin [51] and glimepiride. The classification of the polymers used with FDM 3DP is based on different printing temperatures.

Polymers with printing temperature < 100 °C: HPMC, PVP, Avicel (PH101, PH105), Polyplasdone, Carbopol 794, PEO, Eudragit (E, RL100), PCL, TEC, TCP, and PVA (20–30 K, 83 K, K25).

Polymers with printing temperature 100–150 °C: Eudragit (RS, E, RL, PO, EPO, L100-55, S100, RSPO), PEG (400, 4000, 6000), PEO, Soluplus, PVA, HPC (SSL, L-HPC), HPMC (15 LV), Kollidon (VA64, VA65, CL), Affinisol (15 LV), Kolicot (IR), POLYOX (WSR, N10, WSR, N80), PCL, PLA, and PVP.

Polymers with printing temperature > 150 °C: PVA, PLA, EC(N14), HPC (SL, EF, SSL, LF), PEG (6000, 400, 4000), Eudragit (RL, PO, L100-55, L, RL, EPO, RS 100, RL 100, L100), HPMCAS, PEO (B750), PVA-PEG, PVC-PVA-PEG, cellulose acetate, PVP (K30), Affinisol (15 LV), Kollidon (SR, VA64), Kollicoat IR, and PCL.

Another classification of polymers is based on their release; the following are examples [92]:

Polymers with immediate release: PEG, PVP, Eudragit^®^ EPO, PEO, Kollidon^®^ VA64, and Kollicoat^®^ IR.

Polymers with sustained release: HPMC, TPU, HPC, PLA, PVA, PCL, Eudragit^®^ RL, and Soluplus^®^.

Polymers with delayed release: Eudragit^®^ L, HPMCAS, and Eudragit^®^ S.

Gioumouxouzis et al. studied the capability of FDM 3D printing to formulate a dosage form containing two active pharmaceutical ingredients, metformin and glimepiride. Metformin was mixed with Eudragit^®^ RL to achieve a sustained-release effect, and glimepiride was mixed with polyvinyl alcohol (PVA) for an immediate-release system. The incorporation of the anti-diabetic drugs into two different carriers, forming a bilayer final dosage form, accounted for the distinct release characteristics [51]. Windolf et al. studied the effect of different polymers on the intended sustained release of Levodopa and Benserazide in the treatment of Parkinson’s disease. It was determined that PVA and HPC were not considered because they released the API too fast: HPC 75% Levodopa in 25 min and PVA 75% in 33 min, which was attributed to their high hydrophilicity, formation of a hydrocolloid matrix, swelling, and the eroding properties of the matrix. Therefore, the active ingredients solubilized faster. The active ingredient release using HPMC-AS was too slow, 25% Levodopa in 63 h, so the decision was made for the SR polymer ethylene-vinyl acetate 50% Levodopa in 75 h. In addition, ethylene-vinyl acetate has a lower density than water and gastric fluid, so this property can be essential for a floating, gastro-retentive drug delivery dosage form [67]. They also studied the effect of the percentage of ethylene-vinyl acetate on filament printability; with 28% ethylene-vinyl acetate, the flexibility of the filament was too high, so the printability was poor, the printed objects were not reproducible, and the printing process repeatedly stopped because the filament clogged the nozzle. The percentage of ethylene-vinyl acetate was decreased to 18% to overcome the printability issue [67].

### 6.3. Plasticizers

Plasticizers are among the main excipients used in FDM 3DP printing. Plasticizers are added to the polymer and API to decrease the melting point of the filaments, lower the glass transition temperature, and, therefore, enhance elasticity and workability [93]. This will facilitate printing at a lower temperature, therefore decreasing the risk of API degradation. The most common plasticizers used in literature are 5–30% triethyl citrate, 10% tween 80 [54], 10% citric acid monohydrate [51], 25% sorbitol [50], 20% PEO [94,95], 5% stearic acid [96], or 5% PEG400 [51,97]. Pereira et al. studied the effect of the addition of water as a plasticizer to permit lower processing temperatures and the significance of polypill architecture on drug release. The effect of the addition of water to sorbitol as a plasticizer at a weight equivalent of 20% of the blend enabled the extrusion of the PVA filament at a significantly lower temperature, from 180 °C to 90 °C; the printing temperature was also decreased from 210 °C to 150 °C [73]. Keikhosravi et al., 2020 studied the effect of plasticizers on FDM 3D printing and melt casting as an innovative platform for cardiovascular disease polypill production technique. Increasing the amount of TEC in the filament from 20% to 30% decreased the Young’s modulus of the filament whilst the elongation at break % increased. This indicates that filaments lose their stiffness and brittleness when the amount of TEC is increased. Filaments with low stiffness were not printable (30% TEC), whilst the 20% showed better printability [58].

Zhang et al. also studied the addition of plasticizers to the combi-pill containing tranexamic acid and indomethacin. Plasticizers improved the elasticity of the filament and increased the mechanical suitability of the filament for FDM. Therefore, the addition of Tween 80, PEG 4000, and PEO as plasticizers improved the printability of the filaments, and they were able to sustain the force experienced during FDM feeding and printing [70]. Furthermore, Gioumouxouzis et al. studied filament production by using and evaluating a variety of plasticizers (citric acid monohydrate, triethyl citrate (TEC), PEG 400). The mechanical properties of the produced filaments were improved with the addition of plasticizers [51]. Windolf et al. added 10% mannitol as a plasticizer, as without mannitol addition, the filament became too brittle [67].

### 6.4. Binders

Binders are added to the polymer and API to enhance and improve the unity of the powder mixture during filament fabrication, therefore improving friability and hardness. Hydrophilic binders can also improve the dissolution profiles of hydrophobic API by increasing wettability. Binders are categorized into sugars, synthetic binders, and natural binders [98,99]. Natural binders are inexpensive; examples include gum, acacia, and starch. Synthetic binders are resins, polymers, or oils. Examples of synthetic binders are HPMC, PVC, and MC. Sugars include sucrose, sorbitol, and glucose [100]. Zhang et al. added HPMC as a binder in the combi-pill formulation of tranexamic acid and indomethacin for immediate-release and sustained-release profiles [70].

### 6.5. Fillers

Fillers are materials added to the polymer and API to increase the volume and allow the production of an average-size pill if the quantities of constituents (e.g., API) are low or to increase the density of filaments, which are flexible and have low mechanical properties. Fillers are usually added with binders, as they possess a low binding capacity. Examples of fillers used in the literature are talc [101,102], tri-calcium phosphate 25%, and tribasic phosphate sodium [102]. Sadia et al. added tri-calcium phosphate to between 25% and 50% of the composition of the filament used to produce an antihypertensive polypill. The study concluded that the addition of tri-calcium phosphate allowed the production of a filament with consistent properties and enhanced the viscoelastic properties of the filament [80].

### 6.6. Lubricants

Lubricants are added to filament fabrication, allowing better rheology, improving material flow, reducing friction, and easing the passage through the nozzle of the 3DP. The most commonly used lubricants for FDM 3DP printing are 5% magnesium stearate [36,103], 3% calcium stearate, PEG [104], or 1% fumed silica [67].

### 6.7. Release Modifiers

The main role of adding release modifiers to the polymer and API is to enhance disintegration and dissolution, therefore expediting drug release kinetics. They modify the release of API by fragmenting the tablet into smaller particles, increasing surface area and, therefore, increasing absorption and dissolution. The mechanical characteristics of stiffness and brittleness can be adjusted significantly by changing the type of release modifiers. Furthermore, drug release can be controlled by altering the type of release modifiers. Examples of release modifiers used in polypill production are 20% poly(vinyl alcohol) (PVA), 20% Soluplus^®^, 20% polyethylene glycol (PEG) 6000, 20% Eudragit^®^ RL PO/RS PO, 20% hydroxypropyl methylcellulose (HPMC) K4M/E10M/K100M, and 10–15% Kollidon^®^ vinyl acetate 64 (VA 64)/17PF/30 [57].

### 6.8. Disintegrants

Disintegrants are hydrophilic and swellable in nature; they promote moisture penetration into the tablet, allowing the breakup of the tablet [105,106,107,108]. Examples of release modifiers used in 3DP printing are microcrystalline cellulose [104], xanthan gum [77], sodium lauryl sulfate [109], mannitol [104], croscarmellose sodium, and sodium starch glycolate [109]. Zang et al. added 10–15% carboxymethyl cellulose as a disintegrant, which facilitated the rapid breakdown of tablets into small fragments, resulting in faster dissolution and rapid drug release of tranexamic acid and indomethacin from the combi-pill [73].

### 6.9. Stabilizers

Stabilizers are added in the filament fabrication process to protect the active ingredient from oxidation and hydrolysis. In literature, mannitol and magnesium carbonate are used in the printing of ramipril [110], and titanium dioxide [73]; 1% titanium dioxide was added to the formulation of all individual drug filaments except amlodipine besylate, as titanium dioxide retained its crystalline behavior when it was loaded within the polymeric matrix [73].

## 7. Dosage Forms with More than One API

The interest in research for dosage forms with more than one API “polypill” stems from its potential to revolutionize healthcare by improving medication adherence, enhancing therapeutic outcomes, reducing costs, and simplifying treatment regimens. As researchers continue to explore and refine these formulations, the impact on public health and patient well-being is likely to be significant. Table 1 lists multi-active ingredient 3D-printed dosage forms, inclusive of extrusion and printing particulars. In this review, missing information that could not be imputed or reliably estimated was not filled in. As a result, tables summarizing the data reflect only those entries where complete information was available. Missing data points are indicated by empty cells in the tables, which highlight gaps in the available evidence.

## 8. Characterization

The properties of the final printed dosage form are influenced greatly by the materials used and the design of the printed dosage form, although there are no specific guidelines or tests for 3DP dosage forms and no GMP guidelines. In the literature, there are two categories of characterization with FDM: characterization of the filament before printing and characterization of the final printed dosage form. Filament characterization includes uniform diameter, mechanical property, rheological property, thermal property, and surface properties and external structure. The final dosage form characterization is vast, and no GMP guidelines have been set for 3D dosage forms. In the literature, the final dosage form characterizations include mass, dimensions, uniformity, surface properties, porosity, mechanical properties, solid state, thermal analysis, drug content, disintegration, moisture content, and dissolution studies [113,114]. Table 2 shows the characterization of prepared extruded filament used for the production of polypills through FDM and quality attributes of polypills produced by FDM. The most often found types of characterization of filaments found in the literature were drug content and the measurement of mechanical properties of the filament. All of the literature studied the in vitro dissolution of polypills. Drug content, morphology, SEM, DSC, TGA, and XRD/XPRD were common characterization tests, while FTIR, Raman, TEM, and Micro-CT were the least common tests in the literature. Only one study performed in vivo studies.

The composition of the filament is important for producing high-quality 3D prints. It should have a consistent diameter and good properties to ensure smooth printing. Proper dimensions and properties are necessary for the printing process to work well. Maintaining a uniform filament diameter is crucial for consistent 3D-printed drug forms. The mechanical properties of the filament are essential for effective 3D printing; it needs to be sturdy yet flexible. Various tests can determine the mechanical properties required. The viscosity of the filament impacts printing and material deposition; specific viscosity combinations are necessary for successful printing. Thermal properties play a crucial role in 3D printing. Proper adhesion between layers is needed for high mechanical strength. Differential scanning calorimetry and thermogravimetric analysis can test thermal properties and drug degradation. Surface properties and structure can be observed using a scanning electron microscope. Characterizing the final dosage form is essential for ensuring its properties align with the intended use. Common properties are typically evaluated to ensure quality. Maintaining consistent flow in FDM 3D printing can be challenging. Surface properties can be analyzed using various microscopy techniques; measuring the mechanical properties of 3D-printed dosage forms is important for their integrity. Ideal values are crucial for tablets’ flexibility, hardness, and brittleness. Moisture content in printed formulations affects stability and dissolution. Quality control involves examining raw compounds, filaments, and final printed forms to ensure integrity. Dissolution tests are conducted to determine how the dosage form dissolves in the body. Long-term stability should be maintained for at least a few weeks to prevent API degradation; properties should be evaluated after this period.

## 9. Discussion

FDM in 3D printing of dosage forms is effective, simple, and adaptable for polypills, improving patient outcomes. The benefits of 3DP—such as customized drug release profiles and the ability to integrate multiple drugs into a single dosage form—are consistent across different 3DP methods. The advantages of FDM polypills over traditional manufacturing are highlighted [115]. In terms of demographics, the results align with broader trends observed in 3D-printing technology [28]. Table 3 lists the advantages and disadvantages of FDM 3DP.

One of the primary limitations of this review is the relatively small number of studies available on FDM 3D printing for polypills. The limited volume of research restricts the ability to perform comprehensive analyses and may affect the generalizability of the findings. The small number of studies also reduces the robustness of conclusions and increases the potential for selection bias in the review process. This suggests that practitioners and pharmaceutical developers may not fully exploit the potential benefits of 3D printing for polypills, such as customized drug delivery and improved patient compliance. Expanding the use of FDM 3DP in this domain could enhance the ability to tailor medications to individual needs, thus offering a more patient-centered approach to treatment.

Another limitation of this systematic review is the absence of a pre-registered review protocol. Ideally, a detailed protocol outlining the review methodology, including criteria for study selection, data extraction procedures, and planned analyses, should be developed and registered prior to conducting the review. Also, different studies may use varying terminologies or synonyms for similar concepts. This variability in keyword usage could lead to inconsistencies in identifying relevant literature, potentially affecting the comprehensiveness of the review.

Challenges in 3D printing include filament issues, adhesion problems, and variability affecting polypill quality. FDM faces limitations like drug degradation and filament interactions altering polypill characteristics. Cold extrusion is a potential strategy to avoid thermal degradation and use a variety of polymer materials [102].

Uniformity in the drug–polymer mixture is crucial in cold extrusion for precise dosing. Specialized extruders for 3D printing have limitations in filament options and resolution. The lack of quality control protocols and GMP standards is a concern for FDM printing’s safety and efficacy.

Pharmacopeias lack analysis methods for FDM 3DP final dosage forms. Quality control testing is challenging for personalized 3DP forms due to limited production and patient variations, leading to regulatory concerns. Responsibility for quality control, defective products, and adverse effects is crucial, along with patent regulations for 3D-printed polypills [116].

FDM 3D printing shows promise for polypill fabrication, with innovative API incorporation techniques and exploration of printing parameters. Design improvements in FDM printers and the incorporation of HME technology to enhance efficiency and scale-up production are areas for future research. The creation of a polymer database and the development of filaments for personalized medication are important aspects to consider in FDM 3D-printing investigations [58].

The utilization of 3D-printing technology holds the potential to significantly diminish expenses associated with manufacturing facilities, transportation logistics, warehousing, and obsolescence-related costs. Furthermore, exploring the integration of eco-friendly energy sources to power 3D printers could further bolster the cost-effectiveness of this approach while concurrently mitigating environmental pollution.

The successful implementation of polypills through FDM 3D printing requires collaborative efforts from health authorities and stakeholders to establish quality control standards and regulatory guidelines. Licensing and training standards for pharmacists operating FDM 3D printers are necessary, along with ensuring proper production environments to avoid contamination. Overcoming limitations will lead to the widespread use of FDM 3D printing for tailored dosage forms. Further studies are needed to develop a structured framework for utilizing FDM polypills in pharmaceutical dosage forms.

## Figures and Tables

**Table 1:** List of printed dosage forms with more than one API, inclusive of extrusion and printing details.

Combined API	Polymer(s) %	Other Excipients	HME Extrusion Temp/°C	Screw Speed/rpm	Nozzle Size/mm	Extruder Model	Print Design Software	Printer	Printing Temp/°C	Printing Speed mm/s	Infill %	Nozzle Size	Layer Height	Build Platform Temp °C	Dosage Form and Shape	Reference
Lisinopril dihydrate 5%, indapamide 1.25%, rosuvastatin calcium 5%, amlodipine besylate 2.5% *	60% PVA	Water in filament but dried after extrusion * Sorbitol 25.9%, titanium dioxide 1%	90	35	1.7	Thermo Scientific HAAKE MiniCTW hot melt extruder (Karlsruhe, Germany)	Autodesk^®^ 3ds Max Design 2016 software version 18.0 (Autodesk, Inc., San Francisco, CA, USA)	Makerbot Replicator 2x 3D printer (Makerbot Industries, New York, NY, USA)	150 °C		100	0.4 mm nozzle	166 μm	40 °C	Tablet	[73])
Lisinopril dihydrate 10%, amlodipine besylate 5%, indapamide 2.5%, rosuvastatin calcium 10%	PEG 4000 10%, PEG 400 30% Shell: PLA for immediate release AND PVA for extended release	Lactose monohydrate	Ready PLA/PVA filaments				Autodesk 3ds Max Design 2016 software version 18.0 (Autodesk, Inc., USA)	Makerbot Replicator 2X (Makerbot Industries, LLC, USA)	200 °C					50 °C	Capsule, oval	[72]
Rifampicin (RIF) and isoniazid (ISO) 30%	PEO 70% PLA shell PVA Cap		80	30	1	Twin-screw compounder (DSM, ^®^XPLORE, Amsterdam, The Netherlands)	Comsol Multiphysics (Comsol, Stockholm, Sweden, v5.1),	Dual-nozzle Ultimaker 3 Extended printer (Geldermalsen, The Netherlands)		35 mm/s	100		0.2 mm	60 °C	Unique dual-compartmental dosage unit cylinder	[110]
50% Metformin	35% Eudragit^®^ RL, 10% PLA 5% PEG 400	Citric acid monohydrate, triethyl citrate (TEC)	140	35	1.75	Filabot Original^®^ single-screw extruder (Filabot Inc., Barre, VT, USA) *	AutoCAD 2016^®^ (Autodesk Inc., USA) AutoCAD 2016^®^ (Autodesk Inc., USA)	MakerBot Replicator^®^ 2X 3D printer (MakerBot Inc., NY, USA)	170 °C	70 mm/s, 70 mm/s	100	0.4 mm	0.2	90 °C 90 °C	Tablet, flat cylindrical with smoothed edges (pill-shaped	[51]
2% Glimepiride	80% PVA	15% MANNITOL, 3% CA STEARATE	190	23	1.5	co-rotating twin-screw HAAKE MiniLab^®^ extruder (Thermo Scientific, Waltham, MA, USA) *			205 °C		100		0.2 mm			
Simvastatin 0.5%, aspirin 5%	PEG6000 70%	TEC 20% *, silicon dioxide 3%, glycerin 10%	135 *	16	1.75	UK Noztec desktop filament extruder	Rhinoceros 5 (McNeel & Associates, Seattle, WA, USA	Quantum 2025 desktop printer, Persia 3D printer Co., Tehran, Iran	178 °C	3.5 mm/s	100	0.4 mm	0.3 mm	45 °C	Tablet, half-divided cylindrical shape body	[56]
Enalapril maleate (EM) and hydrochlorothiazide (HCT) EPO: EM: TCP 35:15:50 Eudragit EPO:TEC:HCT:TCP (46.75:3.25:25:25% *w*/*w*) *	Eudragit EPO	Tri-calcium phosphate (TCP), triethyl citrate (TEC)	100	35	1.7	HAAKE MiniCTW hot melt extruder (Thermo Scientific, Karlsruhe, Germany)	Autodesk^®^ 3ds Max Design 2016 software version 18.0 (Autodesk, Inc., USA)	Makerbot Replicator 2× (Makerbot Industries, LLC, USA	135 °C		100			60 °C	Tablet	[53]
Paracetamol (4.3 and 8.2%) or caffeine (4.7 and 9.5%)	PVA		180	15	1.75	Noztek Pro hot melt extruder (Noztek, Shoreham-by-Sea, UK)	AutoCAD 2014R (Autodesk Inc., USA)	MakerBot Replicator 2X (MakerBot Inc, USA).	200 °C	90 mm/s	100				Capsule-shaped tablet, “Caplet”	[68]
Tranexamic acid 10%, indomethacin 17%	Eudragit RL 17%, PEG 4000 10%, PEO 20% *	Tween80 10%, plasticizer 17%	100	100 rpm	1.75	Haake Minilab extruder	Ready PVA filaments Mowiol^®^ 4–88, MW~31.000), were purchased from Sigma-Aldrich, Ann Arbor, MI, USA	Prusa i3 (Mk3S, Prague, Czech Republic	100 °C		10, 30			Room temp, 21 °C		[70]
Lansoprazole, curcumin 20%	HPC and Soluplus	Black seed oil, Capmul MCM, Transcutol P, and Kolliphor ELP	140 *	50 rpm	1.9	Leistritz ZSE 12 HP-PH 12 mm twine-screw corotating extruder	(Dassault Systèmes, Vélizy-Villacoublay, France)	Ultimaker S3, Utrecht, The Netherlands	250 °C *	50 mm/s	100			80 °C	Capsule-shaped tablet, “Caplet”	[57]
Levodopa, Benserazide, and Pramipexole *	PVA, PVP, VA, EVA	Mannitol, fumed silica	100 and 180	20	1.85	Co-rotating twin-screw extruder (Pharmalab HME 16, Thermo Fisher Scientific, Rockford, IL, USA	Fusion 360 (Autodesk, San Rafael, CA, USA)	Prusa 3D printer (Prusa i3 Mk3, Prusa research, Prague, Czech Republic)	150 °C	10 mm/s	100	0.4 mm		70 °C	Various designs *	[67]
Paracetamol and ibuprofen	PLA	Water: glycerol: gelatin	180	15 *	1.75	Noztek Pro hot melt extruder (Noztek, UK)	Autodesk^®^ 3ds Max^®^ Design version 2018 (Autodesk Inc., San Rafael, CA, USA	MakerBot Replicator Experimental 2X dual FDM 3D printer (MakerBot Industries, Brooklyn, NY, USA)	80 °C	50–65 mm/s	25, 50, 75	1.52 mm nozzle size		75 °C	LegoTM-like design	[52]
Hydroxychloroquine, IgG, gp120, and coumarin			160 and 150	100	1.6	HAAKE™ MiniLab II Micro Compounder (ThermoFisher	Simplify3D v3.1 (Simplify3D, LLC, Blue Ash, OH, USA)	lab-developed Cartesian 3D printer	220 °C	15 mm/s	25 to 100	210 micrometer			Intra-vaginal rings	[75]
Nifedipine 20 mg, gliclazide 10 mg, simvastatin 10 mg	HPC (5%), HPMCAS (36.5%),	PEG 4000 (7.5%), magnesium stearate (1%)	NFD 160 °C, SMV at 135 °C, and GLZ at 145 °C	30 rpm	2 mm	Noztek touch single-screw extruder (Shoreham, UK)	Tinkercad^®^	Anycubic Mega Zero	160 °C for NFD, 140 °C for GLZ, and 155 °C	30 mm/s	100	0.4 mm	0.1 mm		Rounded tablets	[111]
Diltiazem, propranolol, and hydrochlorothiazide		PEG 400 (1% *w*/*w*)	175	45 rpm	1.6 mm	Noztek Touch, Noztek England	AutoCAD 2016^®^	MakerBot^®^ Replicator 2X Desktop 3D printer, MakerBot Inc, USA)	190 °C	10 mm/s	100%	0.2 mm	0.2 mm	100 °C	Hollow, Cheerio-shaped dosage forms	[112]

* variable.

**Table 2:** Characterization of prepared extruded filament used for polypill production by FDM 3DP and the multiple API dosage forms.

Characterization	Extruded Filament	Dosage Form	References
Mechanical properties	√		[51,53,67,70,73,75,111,112]
DSC	√	√	[51,53,56,57,68,70,72,73,110,111,112]
TGA	√	√	[51,53,57,68,72,73,110,112]
XRD/XPRD	√	√	[51,53,72,73,111,112]
Drug content	√	√	[51,52,53,56,68,72,73,110,112]
In vitro release studies	√	√	[51,52,53,56,57,67,68,70,72,73,75,110,111,112]
SEM	√	√	[51,52,53,56,57,70,72,73,110,111,112]
FTIR	√	√	[53,70,111,112]
In vivo	√	√	[110]
Micro-CT		√	[57]
Raman		√	[53,68,112]
Stability		√	[56,73]
Morphology		√	[51,53,56,67,70,73,111,112]
TEM		√	[57]
Ex vivo		√	[111,112]

**Table 3:** Advantages vs. disadvantages of FDM 3DP.

Advantages	Disadvantages
Low cost Portable Ease of use High resolution and precision On-demand manufacturing Produce small batches Tailored medication Easy control of constituents Control design and type of dosage form Dosage flexibility and variability Improve patient case management Ease of control of printing parameters No stability issues Opportunity for drug development, testing, and manufacturing Polypill Decreased polypharmacy Increased patient compliance Single-product production pipeline Manufacturing and distribution at the same place	Unsuitable for thermolabile drugs Reduced options of thermoplastic materials with desired properties Low drug loading Not suitable for up-scale production Slower than conventional methods

## Data Availability

Not applicable.

## Abbreviations

3D	Three-dimensional
3PB	Three-Point Bend
AFM	Atomic Force Microscopy
AM	Additive Manufacturing
API	Active pharmaceutical ingredient
ASA	Acetylsalicylic acid
CAD	Computer-Aided Design
CAP	Cellulose acetate phthalate
DDS	Drug Delivery System
DSC	Differential scanning calorimetry
EC	Ethyl cellulose
EM	Enalapril malate
EMA	European Medicines Agency
EVA	Ethylene-vinyl acetate
FDA	Food and Drug Administration
FDM	Fused Deposition Modeling
FT-IR	Fourier-Transform Infrared Spectroscopy
GMP	Good Manufacturing Practice
HCT	Hydrochlorothiazide
HEC	Hydroxyethyl cellulose
HME	Hot Melt Extrusion
HPC	Hydroxypropyl cellulose
HPLC	High-Performance Liquid Chromatography
HPMC	Hydroxypropyl methylcellulose
HPMCAS	Hydroxypropyl methylcellulose acetate succinate
HPMCP	Hydroxypropyl methylcellulose phthalate
MC	Methyl cellulose
NIR	Near Infra-Red hyperspectral imaging technique
OM	Optical Microscopy
PCL	Poly(ε-caprolactone)
PEG	Polyethylene glycol
PEO	Polyethylene oxide
PLA	Polylactic acid
PRISMA	Preferred Reporting Items for Systematic Reviews and Meta-Analyses
PVA	Polyvinyl alcohol
PVP	Polyvinylpyrrolidone
SEM	Scanning Electron Microscopy
TGA	Thermogravimetric analysis
TEM	Transmission Electron Microscopy
UV	Ultraviolet–Visible Spectroscopy
XRD	X-ray diffraction
XRPD	X-ray Powder Diffractometry
